# Sparse Reconstruction Based Robust Near-Field Source Localization Algorithm †

**DOI:** 10.3390/s18041066

**Published:** 2018-04-02

**Authors:** Sen Li, Bing Li, Bin Lin, Xiaofang Tang, Rongxi He

**Affiliations:** College of Information and Science Technology, Dalian Maritime University, Dalian 116026, China; listen@dlmu.edu.cn (S.L.); dlmulibing@163.com (B.L.); linbin@dlmu.edu.cn (B.L.); fangxtang@163.com (X.T.)

**Keywords:** near-filed, direction of arrival, range estimation, impulsive noise, sparse reconstruction, robust correlation

## Abstract

Non-Gaussian impulsive noise widely exists in the real world, this paper takes the α-stable distribution as the mathematical model of non-Gaussian impulsive noise and works on the joint direction-of-arrival (DOA) and range estimation problem of near-field signals in impulsive noise environment. Since the conventional algorithms based on the classical second order correlation statistics degenerate severely in the impulsive noise environment, this paper adopts two robust correlations, the fractional lower order correlation (FLOC) and the nonlinear transform correlation (NTC), and presents two related near-field localization algorithms. In our proposed algorithms, by exploring the symmetrical characteristic of the array, we construct the robust far-field approximate correlation vector in relation with the DOA only, which allows for bearing estimation based on the sparse reconstruction. With the estimated bearing, the range can consequently be obtained by the sparse reconstruction of the output of a virtual array. The proposed algorithms have the merits of good noise suppression ability, and their effectiveness is demonstrated by the computer simulation results.

## 1. Introduction

The source localization problem in array signal processing is an important problem which has a wide range of applications. For example: related topics in radar, sonar, wireless communications, and seismology is to determine the location of the radiation (or reflection) sources by passive sensor array [1,2,3,4]. According to the distance of the source from the array, the localization problem is divided into far-field source localization problem and near-field source localization problem. When the sources are far away from the array, the localization problem in this case is far-field source localization and the signals transmitted by the sources arrive at the array in the form of a plane wave. At this point, the direction of arrival (DOA) needs to be estimated. When the distance between the sources and the array is within the Fresnel region, the localization problem at this time is near-field source localization and the signals transmitted by the sources arrive at the array in the form of a spherical wave. At this time, the DOA and range must be determined. Compared with the DOA estimation in far-field, the position parameter estimation problem of near-field source is relatively complex.

In recent years, near-field source localization problem has caused the concern in the field of signal processing, and some scholars have started to research on this problem. Huang et al. [5] extended the one-dimensional MUSIC method used in the far-field to the two-dimensional MUSIC method to be used in the near-field. The biggest limitation of this method was that it required a two-dimensional spectral peak search and required a large amount of computation. Starer et al. [6] proposed a path-following approach which transformed the two-dimensional search problem into multiple one-dimensional search problems, and finally solved the problem by iterative method. Lee et al. [7] proposed a modified path-following method that replace the path search by known algebraic paths and further reduce the computational complexity of the algorithm. Based on the symmetric subarrays, Zhi et al. [8] applied the generalized ESPRIT algorithm to search the azimuths of each near-field source. Liu Liang et al. [9] used the idea of rank reduction to construct a manifold matrix that was only related to the steering angle parameters and was used to search the near-field source azimuths. References [10,11,12] reconstructed a matrix related only to the azimuth parameters of the sources, and used the MUSIC algorithm to search for the azimuths. After obtaining the source azimuths estimation, the literatures [8,9,10,11,12] searched the range parameter in corresponding directions, which transformed the two-dimensional search into multiple one-dimensional searches, this type of method had a large array aperture loss. Some studies [13,14] estimated the source azimuths by constructing a higher order statistics (HOS) matrix to reduce the array aperture loss. There are also some other near-field parameter estimation methods, such as, the maximum likelihood estimator proposed in [15] and the weighted linear prediction method presented in [16].

With the successful application of sparse signal reconstruction in far-field DOA estimation [17] and the excellent performance of this kind of algorithm in anti-noise ability and snapshot number, more and more scholars also carry out the research on sparse reconstruction based near-field localization methods. Wang et al. [18] proposed a hybrid source localization algorithm based on the sparse representation of cumulants. Tian et al. [19] proposed a joint algorithm of MUSIC and sparse representation for localization of mixed sources with better estimation performance. In [20,21], the covariance matrix of the received signal of the virtual far-field array was constructed and the two-dimensional near-field parameter estimation problem was converted into the reconstruction problem of two one-dimensional sparse signals. Hu et al. [22,23] achieved a sparse estimation of DOA and range by sparse representation of anti-diagonal elements of received signal covariance matrix, which is similar to the method of [20], but with lower computational complexity. In [24,25], by using the sensor-angle distribution to characterize the sensor-dependent phase progression as a function of the source range and its direction, the sensor-dependent spatial frequency signature was estimated by sparse reconstruction techniques, and the results were then mapped back to source range and DOA estimation for the near-filed source localization.

The background noise of the above near-field localization algorithms assumed that it followed Gaussian distribution or at least had a finite second-order statistics, but in the natural environment or many engineering applications, the noise often exhibits non-Gaussian impulsive nature, and often has large amplitudes in a short time [26]. Mathematically, α-stable distribution model can be used to describe this type of noise [27]. Studies have shown that α-stable distribution process does not have the statistics of order greater than the characteristic exponent α(0 < *α* ≤ 2), so the algorithms based on the second-order statistics (SOS) and HOS will degrade or even fail in α-stable distributed noise environment. To this end, a number of articles had been studied, one way is to replace the SOS with fractional lower order statistics (FLOS), such as the fractional lower order correlation (FLOC) and the phased fractional lower order correlation (PFLOC) [28,29]. However, FLOS needs to know the characteristic exponent of the α-stable distribution in advance, which is difficult to obtain in practice. Another way is to replace the SOS with the robust second-order statistics. For example, the secondorder correlation was replaced by a robust correlation, nonlinear transform correlation (NTC) [30,31], which did not need to know the characteristic exponent of the α-stable distribution in advance. To solve the localization problem of near-field sources in the impulsive noise, Wang et al. [32] constructed matrixes by using FLOS and estimated position parameter of near-field sources by rooting method. By combing the concept of PFLOC and the GESPRIT method in [8], Qiu et al. [33] proposed a new search-free method for near-field source localization under impulsive noise, referred to as the search-free PFLOC-based GESPRIT. We studied the near-field localization problem based on sparse reconstruction of the constructed far-field approximate FLOC vector by exploring the symmetrical characteristic of the array under impulse noise environment for the first time in [34]. This paper is on this basis to do further research on this issue. In this paper, to avoid estimating the characteristic exponent α of the impulsive noise before using the FLOC statistics, we define a robust correlation vector, NTC vector, which is just like the FLOC vector that is in relation with the DOA only, then the bearings can be estimated based on the sparse reconstruction of the FLOC vector or NTC vector. With the estimated bearings, the range can consequently be obtained by the sparse reconstruction of the output of a virtual array. Lots of simulation experiments are made in this paper to demonstrate that the proposed algorithms have the merits of good noise suppression ability.

## 2. α-Stable Distribution

α-stable distribution is the only kind of distribution satisfying the generalized central limit theorem. Compared with the Gaussian distribution, the α-stable distribution has a thicker statistical tail, so it has significant impulse characteristics in time domain. There is no closed expression for the probability density function of the α-stable distribution, but the characteristic function can be used to facilitate the representation of the α-stable distribution

(1) $$\phi \left(t\right)=e^{\left\{jat-\gamma {\left|t\right|}^{\alpha }\left[1+j\beta \mathrm{sgn}\left(t\right)\varpi \left(t,\alpha \right)\right]\right\}}$$

where

(2) $$\varpi (t,a)=\left\{ \begin{array}{l} \tan\frac{\pi \alpha }{2},\;\mathrm{if}\;\alpha \neq 1\\ \frac{2}{\pi }\log|t|,\;\mathrm{if}\;\alpha =1 \end{array}\right.$$

(3) $$\mathrm{sgn}(t)=\left\{ \begin{array}{l} t/|t|,\;\mathrm{if}\;t\neq 0\\ 0,\;\mathrm{if}\;t=0 \end{array}\right..$$

It can be seen that the characteristic function of the α-stable distribution is determined by four parameters: α(0 < *α* ≤ 2) is the characteristic exponent, which describes the impulsive degree of the distribution. The smaller the α is, the thicker the corresponding distribution tail is, the more significant impulsive the signal is; β(−1 < *β* < +1) is the symmetry parameterand that β=0 corresponds to the symmetric distribution, abbreviated as symmetry α-stable (SαS) distribution; γ(γ≥0) is the dispersion which is similar to the variance of Gaussian distribution; a(−∞<a<+∞) is location parameter, for the SαS distribution it represents the median or the mean. When α=2 and β=0, the characteristic function is the same as that of Gaussian distribution, that is, the Gaussian distribution is a special case of the α-stable distribution. A very important characteristic of the α-stable distribution is that it does not have a finite two order statistics and higher order statistics.

## 3. Problem Formulation

### 3.1. Signal Model

**Figure 1:**
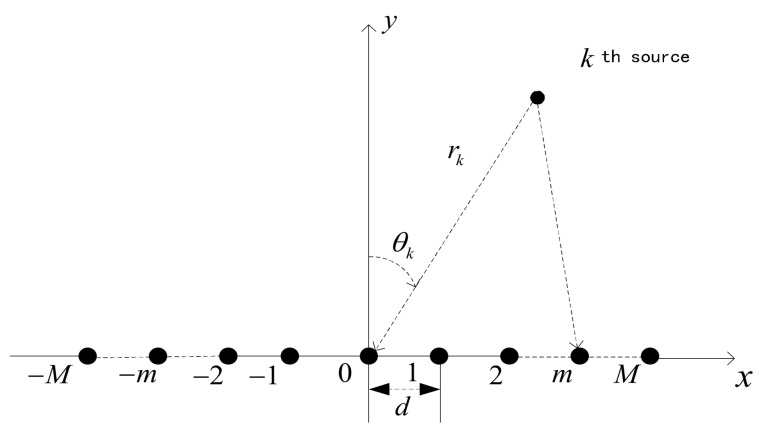
Near-field ULA array.

Consider the case of *K* independent narrowband sources $s_{1}\left(t\right),s_{2}\left(t\right),\cdots ,s_{K}\left(t\right)$ be in the near-field of a symmetric uniform linear array (ULA) with N=2M+1 isotropic sensors as illustrated in Figure 1. With the array center being the phase reference point, the signal received by the *m*th sensor at time *t* can be expressed as

(4) $$x_{m}\left(t\right)=\sum_{k=1}^{K}s_{k}\left(t\right)A_{mk}+n_{m}\left(t\right)=\sum_{k=1}^{K}s_{k}\left(t\right)e^{\left(j\tau_{mk}\right)}+n_{m}\left(t\right),m=-M,\cdots ,M$$

where $n_{m}(t)$ is the additive noise, and $\tau_{mk}$ is phase shift related with the *k*th source signal’s propagation time delay between phase reference point and sensor *m*. By Fresnel approximation, it can be given by

(5) $$\tau_{mk}\approx w_{k}m+\phi_{k}m^{2}$$

$w_{k}$ and $\phi_{k}$ with the following form

(6) $$w_{k}=-\frac{2\pi d}{\lambda }\sin\theta_{k},\;\phi_{k}=\frac{\pi d\cos^{2}\theta_{k}}{\lambda r_{k}}$$

Herein, λ is the wavelength, d is the interspacing,$\left(\theta_{k},r_{k}\right)$ represent the DOA and range parameters of the *k*th source.

The signal model in Equation (4) can be concisely expressed as

(7) X(t)=A(θ,r)S(t)+N(t)

where

(8) $$\begin{array}{c} X\left(t\right)={\left[x_{-M}\left(t\right),\cdots ,x_{0}\left(t\right),\cdots ,x_{M}\left(t\right)\right]}^{T}\\ S\left(t\right)={\left[s_{1}\left(t\right),s_{2}\left(t\right),\cdots ,s_{K}\left(t\right)\right]}^{T}\\ N\left(t\right)={\left[n_{-M}\left(t\right),\cdots ,n_{0}\left(t\right),\cdots ,n_{M}\left(t\right)\right]}^{T}\\ A\left(\theta ,r\right)=\left[a\left(\theta_{1},r_{1}\right),a\left(\theta_{2},r_{2}\right),\cdots ,a\left(\theta_{K},r_{K}\right)\right]\\ a\left(\theta_{k},r_{k}\right)={\left[\exp\left(j\left(\left(-M\right)w_{k}\right)+M^{2}\phi_{k}\right),\cdots ,1,\cdots ,\exp\left(j\left(Mw_{k}+M^{2}\phi_{k}\right)\right)\right]}^{T} \end{array}$$

The purpose is to jointly estimate the DOAs $\theta_{1},\theta_{2},\cdots ,\theta_{K}$ and range parameters $r_{1},r_{2},\cdots ,r_{K}$ for multiple near-field sources from the received array data X=[X(1),X(2),⋯,X(L)] where *L* is the number of snapshots. Assume the source signals are statistically mutually independent with zero mean and the noises $n_{m}(t)$ are complex isotropic Gaussian distributed random processes and are independent of the source signals, the SOCSR algorithm proposed in [23] localized the near-field sources by solving two spare reconstruction problems of the second order correlation vector of the array received signal. When the noises are complex isotropic SαS distributed random processes, the performance of the SOCSR algorithm will degrade since SαS distribution does not have finite SOS. This fact will be verified by the simulations in Section 5. In order to inhibit the influence of the α-stable impulsive noise, this paper proposes two new near-field sources localization algorithms by solving the spare reconstruction problem of the FLOC and NTC vector of the array received signal which is referenced as fraction lower order correlation-based sparse reconstruction method (FLOCSR) algorithm and nonlinear transform correlation-based sparse reconstruction method(NTCSR) algorithm.

### 3.2. FLOC Matrix of the Array Received Signal

The spatial fraction lower order cross correlation between the *m*th and *n*th sensor can be defined as [28,29]

(9) $$\begin{array}{ll} c_{FLOC}\left(m,n\right) & =E\left\{x_{m}\left(t\right)x_{n}{\left(t\right)}^{<p-1>}\right\}=E\left\{x_{m}\left(t\right){\left|x_{n}\left(t\right)\right|}^{p-2}x_{n}^{*}\left(t\right)\right\}\\ & =\sum_{k=1`}^{K}e^{j(w_{k}m+\phi_{k}m^{2})}\Lambda_{kk}e^{-j(w_{k}n+\phi_{k}n^{2})}+\xi \delta \left(m-n\right)\\ & =\sum_{k=1}^{K}A_{mk}\Lambda_{kk}A_{nk}^{*}+\xi \delta_{mn} \end{array}$$

where

$$\Lambda_{kk}=E\left\{s_{k}\left(t\right){\left|\sum_{q=1}^{M}s_{q}\left(t\right)+n_{n}(t)\right|}^{p-2}{\left(\sum_{q=1}^{M}s_{q}\left(t\right)+n_{n}(t)\right)}^{*}\right\}$$

$$\xi =E\left\{n_{n}\left(t\right){\left|\sum_{q=1}^{M}s_{q}\left(t\right)+n_{n}\left(t\right)\right|}^{p-2}{\left(\sum_{q=1}^{M}s_{q}\left(t\right)+n_{n}\left(t\right)\right)}^{*}\right\}$$

In order to ensure $c_{FLOC}\left(m,n\right)$ is a finite value, the value of p needs to be less than the characteristic exponent α of the impulsive noise which is difficult to estimate in some practical applications.

The FLOC matrix of the array received signal can be formulated as

(10) $$C_{FLOC}=A\left(\theta ,r\right)\Lambda A^{H}\left(\theta ,r\right)+\xi I$$

where $\Lambda =diag\left[\Lambda_{11},\Lambda_{22},\cdots \Lambda_{KK}\right]$ and *I* is the identity matrix.

### 3.3. NTC Matrix of the Array Received Signal

In order to avoid estimating the characteristic exponent α of the impulsive noise in practical applications, a robust correlation, the nonlinear transform correlation (NTC), between the *m*th and *n*th sensor is defined as follows [30,31]

(11) $$c_{NTC}\left(m,n\right)=E\left\{\frac{x_{m}\left(t\right){x_{n}}^{*}\left(t\right)}{\left|x_{m}\left(t\right)x_{n}\left(t\right)\right|+\delta^{2}}\right\}$$

where δ≥1 is called scale factor. It has been proved that $c_{NTC}\left(m,n\right)$ is bounded and then the NTC matrix of the array received signal can be estimated.

## 4. Proposed Two Step Estimation Method

The FLOC $c_{FLOC}\left(m,n\right)$ and NTC $c_{NTC}\left(m,n\right)$ can be unified written as the robust correlation $c_{x}\left(m,n\right)$ and the corresponding matrix form can be written as C. When m=−n the robust correlation $c_{x}\left(m,n\right)$ is independent of the parameter $\phi_{k}$. This means that by exploiting the robust correlation between the symmetric sensors, we can transform the original two-dimensional (DOA and range) estimation problem into a one-dimensional (DOA) estimation problem. Stacking Equation (9) or Equation (11) for the symmetric sensors, we can build a virtual far-field model

(12) $$c_{x}=A_{w}\left(\theta \right)\Lambda_{w}$$

where $c_{x}={\left[c_{x}(-M,M),\ldots ,c_{x}(-1,1),c_{x}(1,-1),\ldots ,c_{x}(M,-M)\right]}^{T}\in C^{2M\times 1}$ and $\Lambda_{w}={\left[\Lambda_{11},\Lambda_{22},\cdots ,\Lambda_{KK}\right]}^{T}$ is the received signal vector and source signal vector of the virtual far-field array, the manifold matrix of the virtual far-field array can be expressed as $A_{w}\left(\theta \right)=\left[a_{w}\left(\theta_{1}\right),a_{w}\left(\theta_{2}\right),\cdots ,a_{w}\left(\theta_{K}\right)\right]\in C^{2M\times K}$ with the virtual array steering vector $a_{w}\left(\theta_{k}\right)=[e^{-j2Mw_{k}},\cdots ,e^{-j2w_{k}},e^{j2w_{k}}\cdots ,{e^{j2Mw_{k}}]}^{T}\in C^{2M\times 1}$.

### 4.1. Step-1: DOA Estimation

The virtual far-field array received signal vector $c_{x}$ can be sparsely represented in a redundant basis. Define a set $\hat{\theta }=\left[{\hat{\theta }}_{1},{\hat{\theta }}_{2},\cdots ,{\hat{\theta }}_{N_{\theta }}\right]$ which denotes potential DOAs of interest sources and assume that the true DOAs are exactly on this set. The number of the potential DOAs $N_{\theta }$ should be much greater than K which is the number of sources. Define the overcomplete basis $A_{w}\left(\hat{\theta }\right)=\left[a_{w}\left({\hat{\theta }}_{1}\right),a_{w}\left({\hat{\theta }}_{2}\right),\cdots ,a_{w}\left({\hat{\theta }}_{N_{\theta }}\right)\right]$ and the potential source signal vector $v={\left[v_{1},v_{2},\cdots ,v_{N_{\theta }}\right]}^{T}$. As a result $c_{x}$ can be rewritten as the following form

(13) $$c_{x}=A_{w}\left(\hat{\theta }\right)v$$

It can be seen that the elements of vector v have K nonzeros, that is $v_{k}=\Lambda_{ii}$ if ${\hat{\theta }}_{k}=\theta_{i},i=1,\cdots ,K$. Hence the DOA estimation problem can be reduced to finding the nonzero elements of the vector v. Since v is sparse, so it can be estimated by solving the following sparse reconstruction problem

(14) $$\begin{array}{ccc} \min{\left\|v\right\|}_{1} & s.t. & {\left\|c_{x}-A_{w}\left(\hat{\theta }\right)v\right\|}_{2}\leq \varepsilon_{1} \end{array}$$

where $\varepsilon_{1}$ is a parameter which means how much of the error we wish to allow and plays an important role in the algorithm performance.

### 4.2. Step-2: Range Estimation

Given a DOA ${\bar{\theta }}_{k}(k=1,\cdots ,K)$ which is estimated in Step1, the robust correlation matrix of the near-filed received signal can be written as

(15) $$C_{{\bar{\theta }}_{k}}=A\left({\bar{\theta }}_{k},r\right)\Lambda A^{H}\left({\bar{\theta }}_{k},r\right)+\xi I$$

where the manifold matrix $A\left({\bar{\theta }}_{k},r\right)=\left[a\left({\bar{\theta }}_{k},r_{1}\right),a\left({\bar{\theta }}_{k},r_{2}\right),\cdots ,a\left({\bar{\theta }}_{k},r_{K}\right)\right]$. Applying the vectorization operator on Equation (15), we have

(16) $$y_{{\bar{\theta }}_{k}}=vect\left(C_{{\bar{\theta }}_{k}}\right)=B_{{\bar{\theta }}_{k}}(r)\Lambda_{w}+\xi vect\left(I\right)\in C^{N^{2}\times 1}$$

(17) $$B_{{\bar{\theta }}_{k}}(r)=\left[a^{*}\left({\bar{\theta }}_{k},r_{1}\right)⊗a\left({\bar{\theta }}_{k},r_{1}\right),\cdots ,a^{*}\left({\bar{\theta }}_{k},r_{k}\right)⊗a\left({\bar{\theta }}_{k},r_{k}\right)\right]\in C^{N^{2}\times K}$$

where ⊗ denotes Kronecker product. It is interesting to see that $y_{{\bar{\theta }}_{k}}$ in Equation (16) can also be regarded as output of the virtual far-filed array where $B_{{\bar{\theta }}_{k}}(r)$, $\Lambda_{w}$ and ξvect(I) are the virtual manifold matrix, equivalent source signal vector, and equivalent noise vector, respectively. Notice that the vector ξvect(I) has only N nonzero elements, then these elements of $y_{{\bar{\theta }}_{k}}$ corresponding to these positions of nonzero elements in ξvect(I) can be removed and the rest N(N−1) entries of $y_{{\bar{\theta }}_{k}}$ corresponding to these positions of zeros elements in ξvect(I) can be preserved. Then, the virtual far-field array output vector processed by this operation can be formulated as

(18) $$y_{{\bar{\theta }}_{k}}^{\prime }=B_{{\bar{\theta }}_{k}}^{\prime }(r)\Lambda_{w}\in C^{N\left(N-1\right)\times 1}$$

where $B_{{\bar{\theta }}_{k}}^{\prime }(r)\in C^{N(N-1)\times K}$ is the new manifold matrix obtained by removing the rows of matrix $B_{{\bar{\theta }}_{k}}(r)$ which are corresponding to these positions of nonzero elements in ξvect(I). This elimination operation can further reduce the effect of the impulsive noise.

Using a similar approach as in Step-1, the virtual received signal vector $y_{{\bar{\theta }}_{k}}^{\prime }$ can be sparsely represented as the following form

(19) $$y_{{\bar{\theta }}_{k}}^{\prime }=B_{{\bar{\theta }}_{k}}^{\prime }(\hat{r})p$$

where $B_{{\bar{\theta }}_{k}}^{\prime }(\hat{r})\in C^{N(N-1)\times N_{r}}$ is the overcomplete basis on a set $\hat{r}=\left[{\hat{r}}_{1},{\hat{r}}_{2},\cdots ,{\hat{r}}_{N_{r}}\right]$. $N_{r}$ is the number of the potential sources on the direction of ${\bar{\theta }}_{k}$ and should be much greater than the number of real sources $N_{r_{k}}$ on the direction of ${\bar{\theta }}_{k}$, $p={\left[p_{1},p_{2},\cdots ,p_{N_{r}}\right]}^{T}$ is the potential source signal vector that have $N_{r_{k}}$ nonzeros, that is $p_{j}=\Lambda_{k_{i}k_{i}}$ if ${\hat{r}}_{j}=r_{k_{i}}\left(i=1,\cdots ,N_{r_{k}}\right)$. Hence the range parameter estimation problem can be resolved by finding the nonzero elements of vector p, which can be estimated by solving the sparse reconstruction problem given by

(20) $$\begin{array}{ccc} \min{\left\|p\right\|}_{1} & s.t. & {\left\|y_{{\bar{\theta }}_{k}}^{\prime }-B_{{\bar{\theta }}_{k}}^{\prime }(\hat{r})p\right\|}_{2}^{}\leq \varepsilon_{2} \end{array}$$

where $\varepsilon_{2}$ is also a parameter which means how much of the error we wish to allow and plays an important role in the algorithm performance.

## 5. Simulation Results

In order to verify that our proposed FLOCSR and NTCSR algorithms have a more noise-rejection ability than the SOCSR algorithm in the α–stable impulsive noise environment, we conduct a series of numerical experiments under a variety of simulation conditions. In order to solve the convex optimization problem given in Equations (14) and (20), the software package CVX [35] is used. Considering an N=15 element ULA, the separation distance between the elements is λ/4. Uniform sampled in the angular space $\left[-90^{o},90^{o}\right]$ at $1^{o}$ interval and near-field range scope [0.1λ,10λ] at 0.1λ interval, that is $N_{\theta }=181$ and $N_{r}=100$.

As the characteristic of the α-stabledistribution makes the use of the standard SNR meaningless, a new SNR measure, generalized signal-to-noise ratio (GSNR) is defined as [27]

(21) $$\mathrm{GSNR}=10\log_{10}\frac{\sigma_{s}^{2}}{\gamma }$$

where $\sigma_{s}$ is the variance of the signal.

Two performance criteria are used to assess the performance of the algorithms. The first one is the probability of success. A successful simulation is defined if angle difference between the estimated DOA and the real DOA is less than $3^{o}$ and distance difference between the estimated range and the real range is less than 0.3λ for all incident sources. The probability of success is defined as the ratio of the number of successful simulations to the total number of Monte Carlo simulations. Another criterion that used to assess the performance of the algorithms is the average root mean square error (RMSE) defined as

(22) $$RMSE=\frac{1}{K}\sum_{l=1}^{K}\sqrt{\frac{1}{N_{ok}}\sum_{i=1}^{N_{ok}}{\left({\bar{x}}_{l}(i)-x_{l}\right)}^{2}}$$

where $x_{l}$ is the real value of the DOA or the range parameter, ${\bar{x}}_{l}(i)$ is the *i*th estimation value of $x_{l}$ and $N_{ok}$ is the number of successful simulations.

### 5.1. Simulation 1

**Figure 2:**
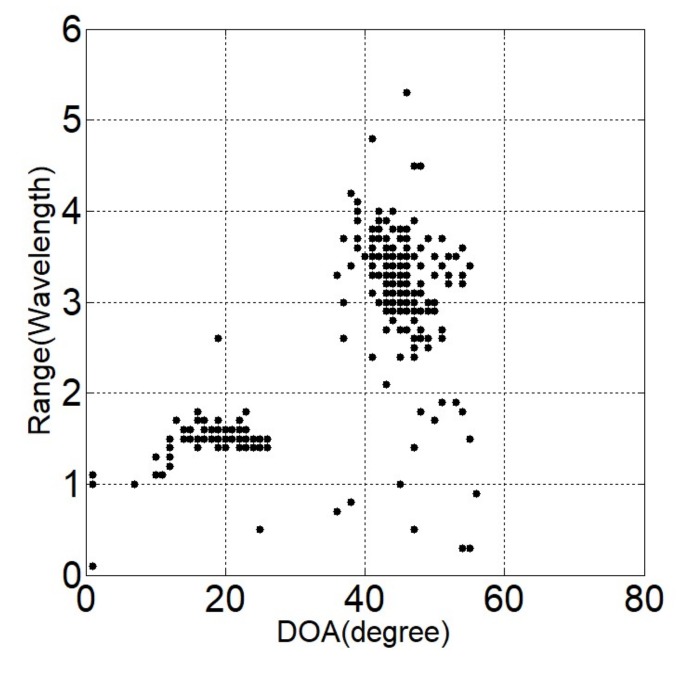
Simulation results of the SOCSR algorithm.

**Figure 3:**
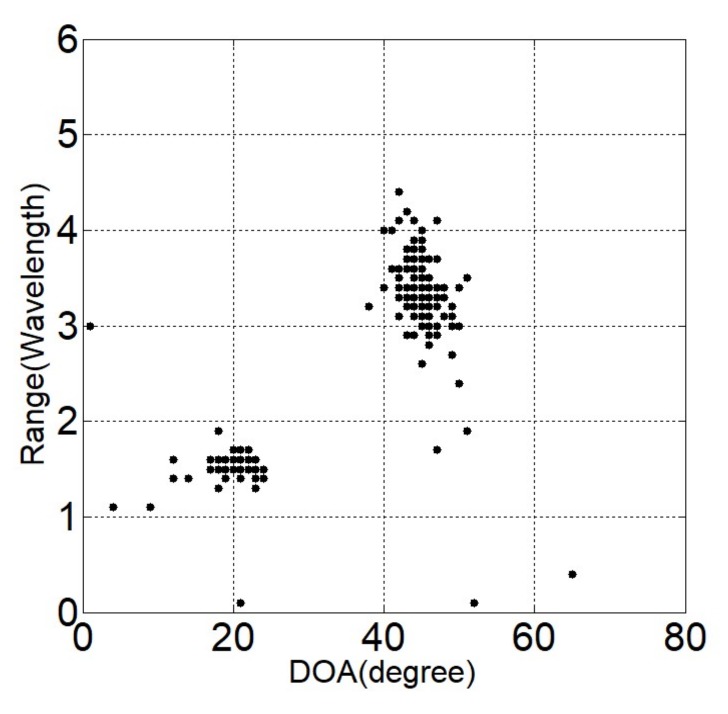
Simulation results of the FLOCSR algorithm.

**Figure 4:**
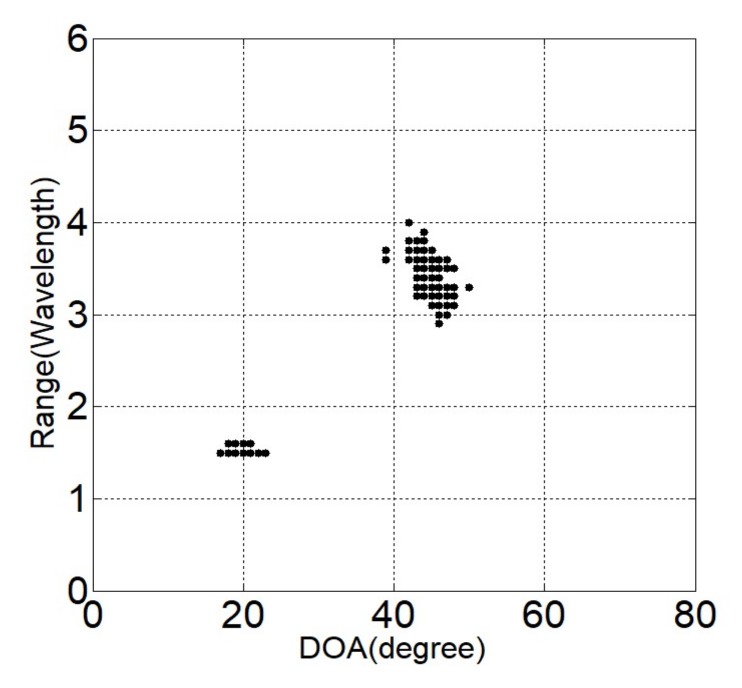
Simulation results of the NTCSR algorithm.

Two independent Gaussian distribution sources with equal power in the near-field region at locations $\left(\theta_{1},r_{1}\right)=(20^{o},1.5\lambda )$ and $\left(\theta_{2},r_{2}\right)=(45^{o},3.6\lambda )$ are considered. Suppose the noise is modeled to be SαS distributed with α=1.5. The generalized SNR is set to be GSNR = 5 dB and the number of snapshots is *L* = 500. Two-hundred Monte-Carlo simulations are performed individually for each method. Figure 2, Figure 3 and Figure 4 give the estimated locations of the SOCSR, FLOCSR, and NTCSR algorithm. It can be seen that the simulation results of the SOCSR algorithm are scattered around the real location, whereas the simulation results of the FLOCSR and NTCSR algorithms are closely gathered near the real location, especially the NTCSR algorithm.

### 5.2. Simulation 2

**Figure 5:**
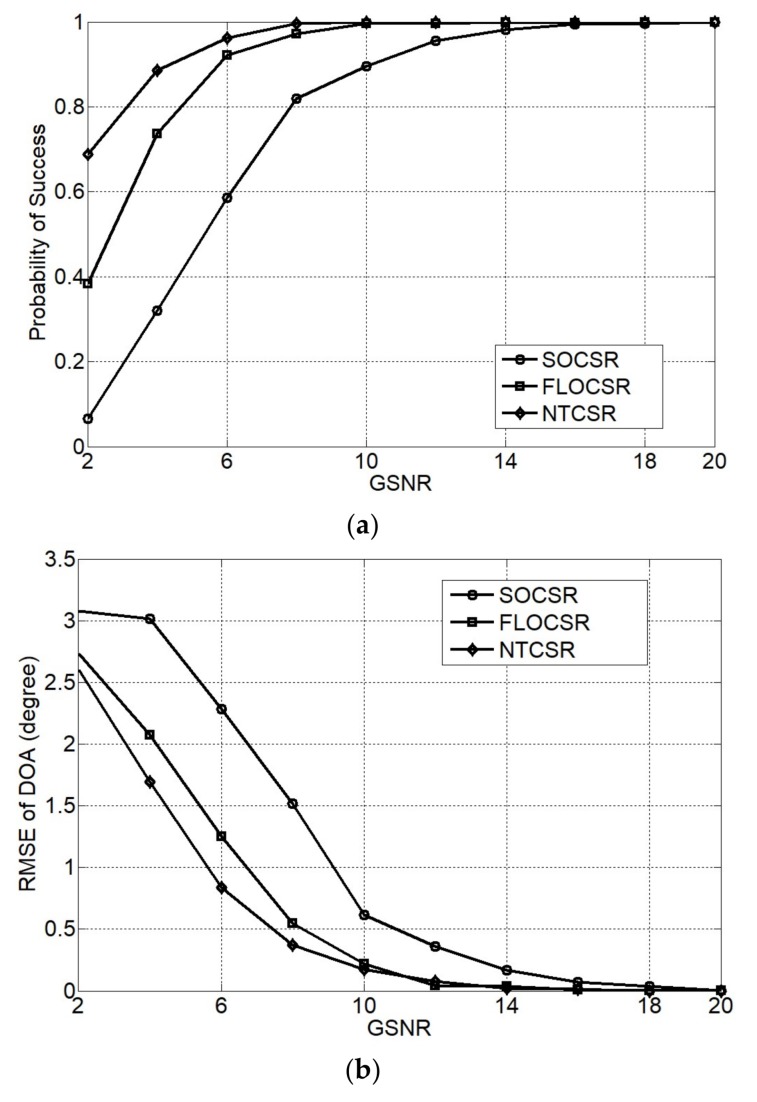

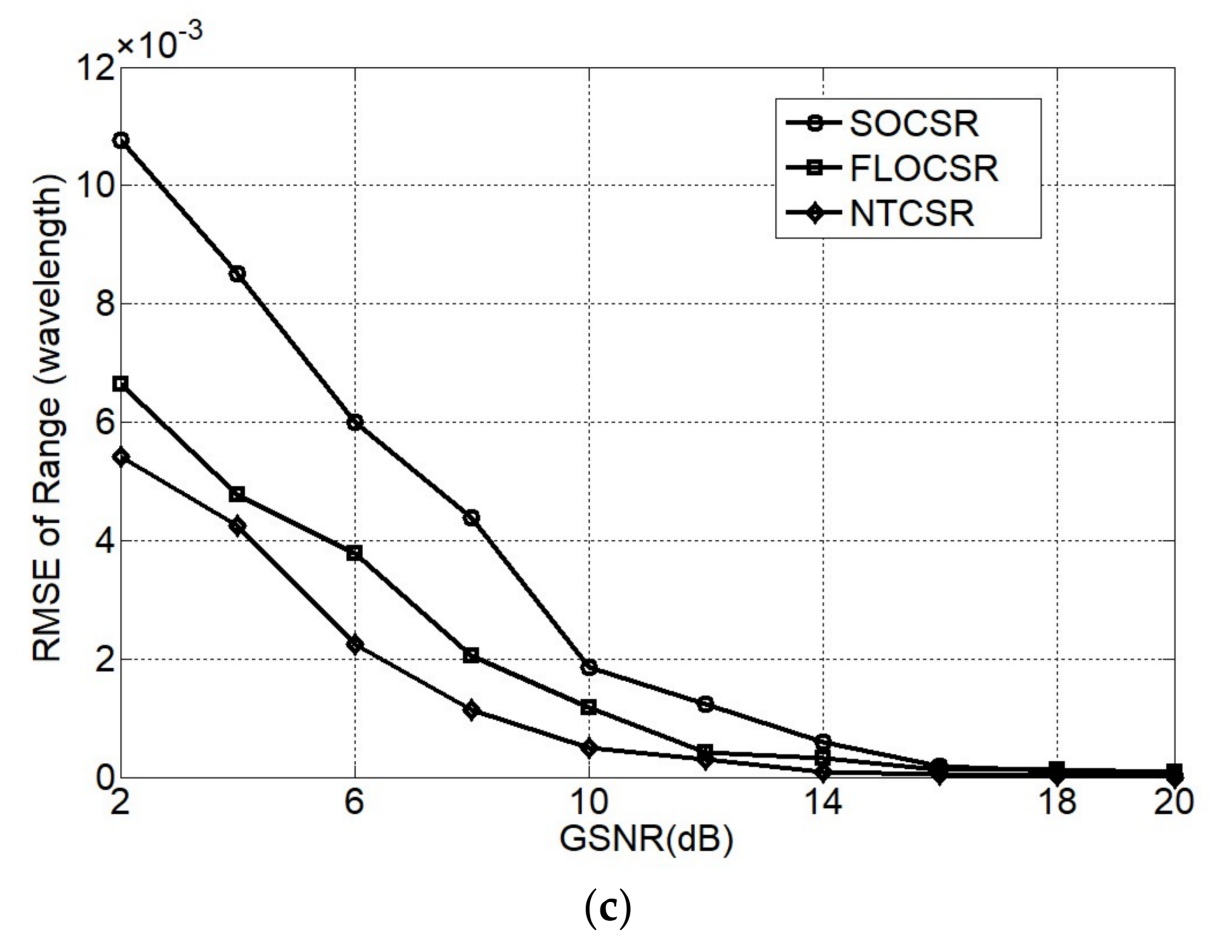
Performance as a function of GSNR (**a**) probability of success; (**b**) RMSE of DOA; and (**c**) RMSE of range.

In this simulation the locations of two independent Gaussian distribution sources with equal power are set as $\left(\theta_{1},r_{1}\right)=(20^{o},0.2\lambda )$ and $\left(\theta_{2},r_{2}\right)=(45^{o},1.4\lambda )$. The characteristic exponent of the α-stable noise is fixed at α=1.5 and thenumber of snapshots is *L* = 1000. Figure 5 shows the performance of the three algorithms for various GSNRs ranging from 2 dB to 20 dB. We see that probability of success of all algorithms improve with the increase of GSNR, and the proposed FLOCSR and NTCSR algorithms outperform the SOCSR algorithm. For example, when GSNR = 6 dB, the probability of success of SOCSR algorithm is only 58% whereas the probability of success of FLOCSR and NTCSR method are all above 90%. The RMSE of DOA and range of all algorithms decrease with the increase of GSNR but the proposed FLOCSR and NTCSR algorithms have a less value than the SOCSR algorithm at the same GSNR. That is to say, the proposed FLOCSR and NTCSR methods have a better estimation accuracy and precision than SOCSR algorithm. From the simulation results, we can also see that the performance of the NTCSR algorithm is better than that of the FLOCSR algorithm, indicating that the nonlinear transform correlation has a better ability to suppress impulse noise compared with the fraction lower order correlation.

### 5.3. Simulation 3

**Figure 6:**
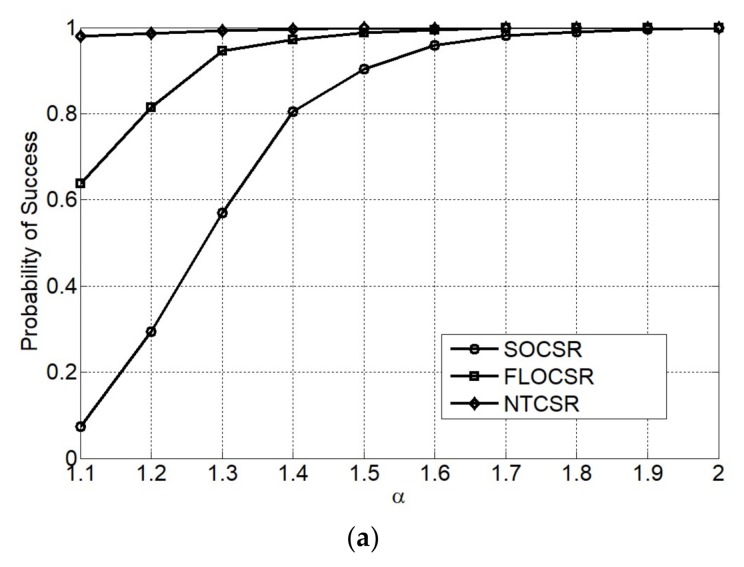

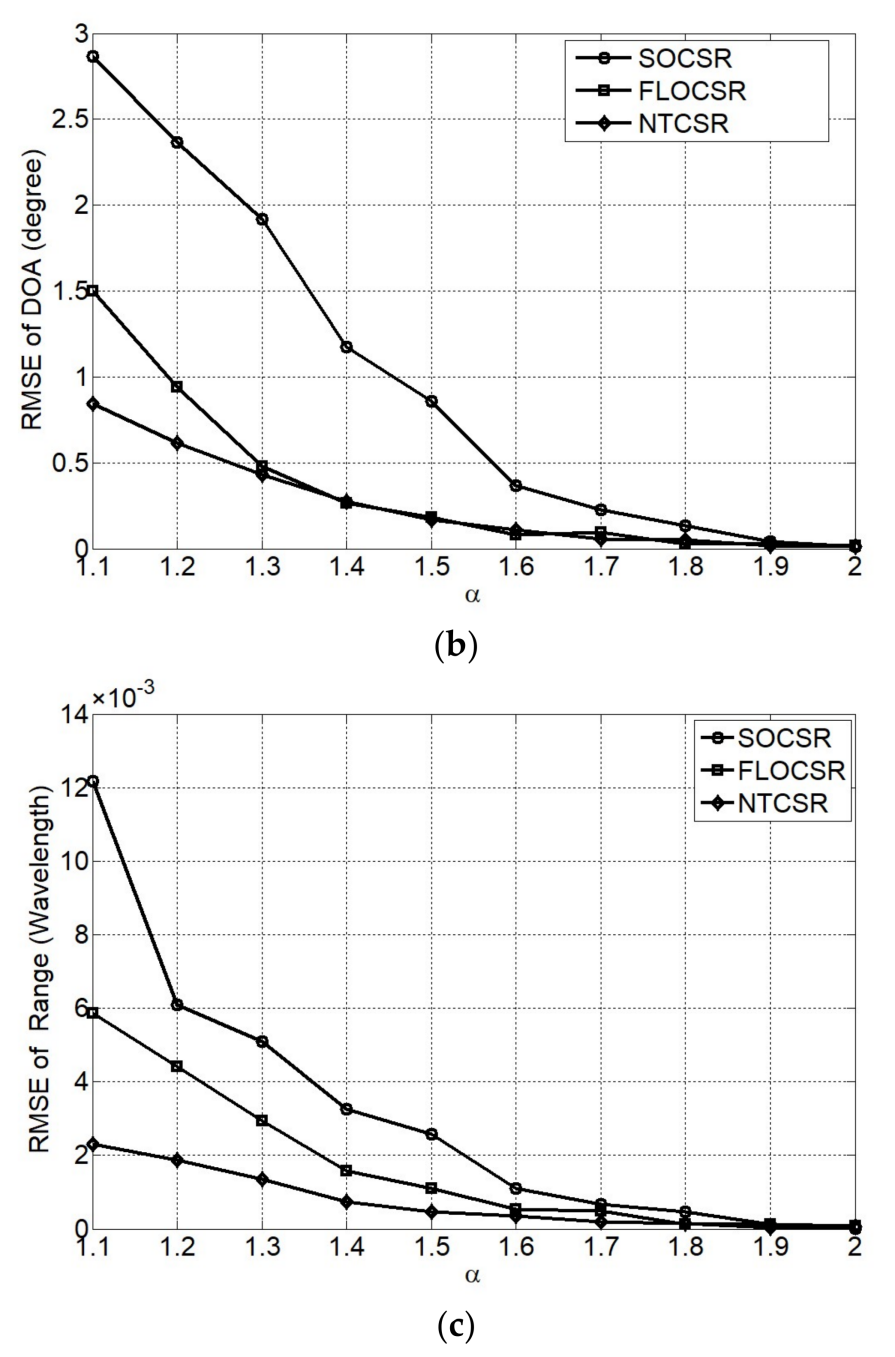
Performance as a function of characteristic exponent α (**a**) probability of success; (**b**) RMSE of DOA; and (**c**) RMSE of range.

Figure 6 plots the performance of the three algorithms varying with different values of the characteristic exponent of the α-stable impulsive noise. The simulation environment is as same as Simulation 2 except that the GSNR is kept at 10 dB. As shown in Figure 6, our proposed FLOCSR and NTCSR algorithms demonstrate their performance enhancement over SOCSR algorithm in the sense of the probability of success and RMSE under the highly impulsive noise environment. In particular, the performance of NTCSR algorithm which has a good ability to suppress impulse noise is more outstanding when in the strong impulse noise environment with characteristic exponent α=1.1, the probability of success is almost 1 and the lowest RMSE.

### 5.4. Simulation 4

**Figure 7:**
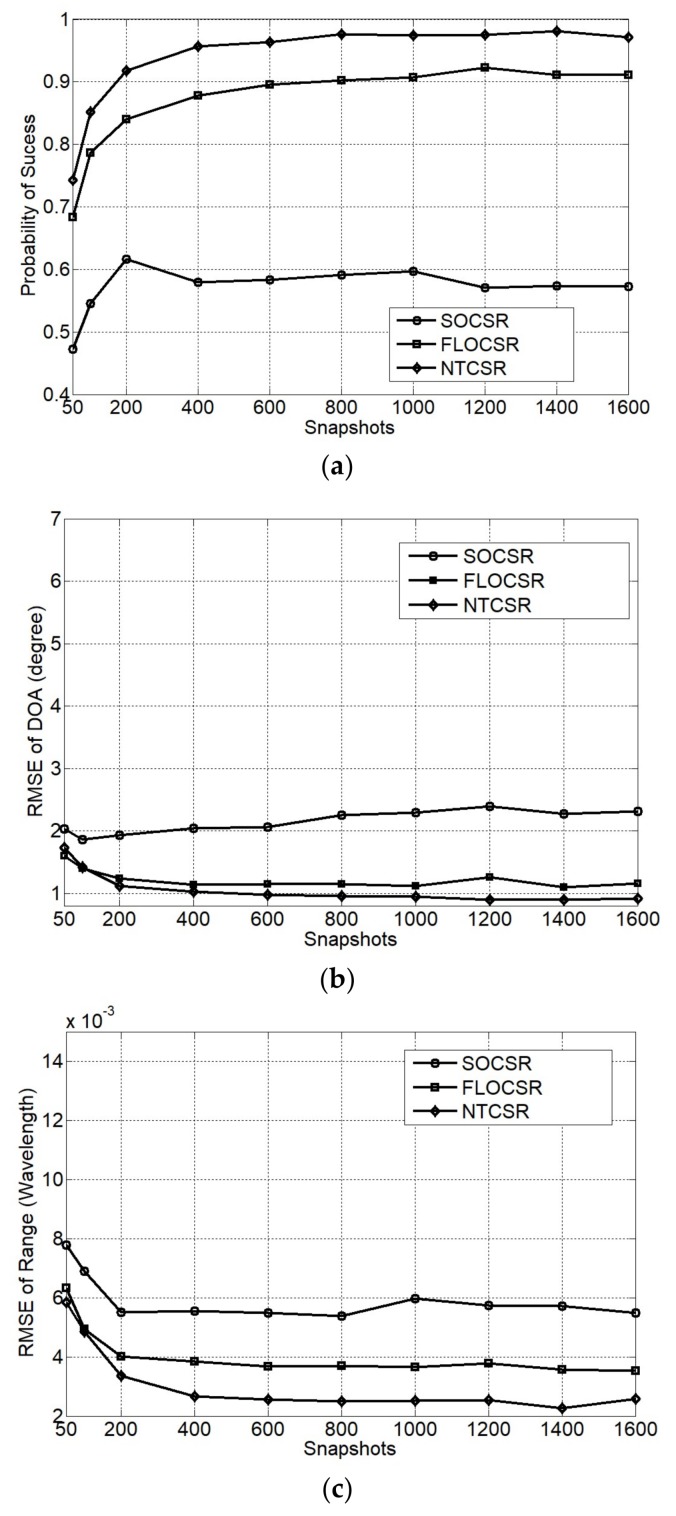
Performance as a function of snapshots (**a**) probability of success; (**b**) RMSE of DOA; and (**c**) RMSE of range.

In this experiment, the simulation environment is accordance with Simulation 2 except for GSNR = 6 dB and the number of snapshots is varied from 50 to 1600. Figure 7 shows the relationship between the performance of the three algorithms and the number of snapshots. We can observe that as the number of snapshots increase all algorithms exhibit a decrease in RMSE and an increase in the probability of success. However when the number of snapshots is more than 400, the increased performance caused by the snapshots is not obvious. Nevertheless, our proposed FLOCSR and NTCSR algorithms produce lower RMSE and higher probability of success compared to the SOCSR algorithm when the same number of snapshots is used.

### 5.5. Simulation 5

**Figure 8:**
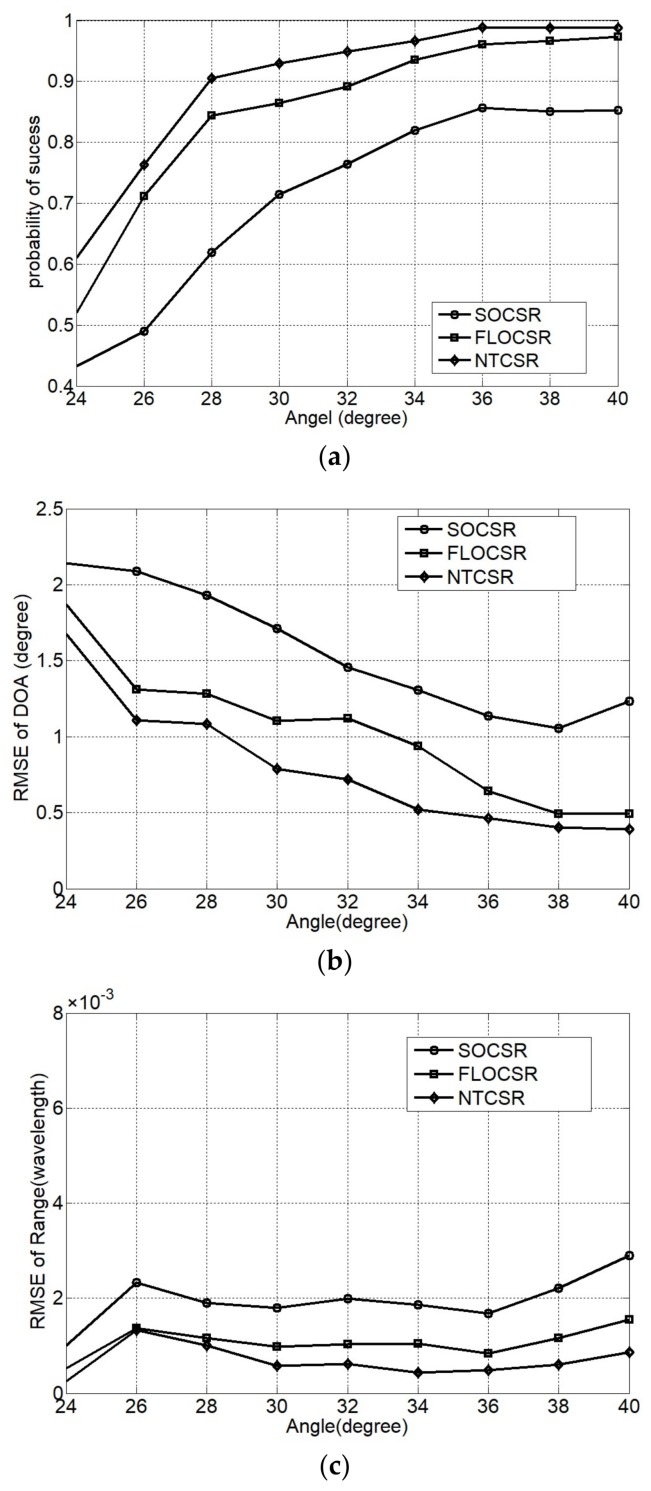
Performance as a function of angle separation (**a**) probability of success; (**b**) RMSE of DOA; and (**c**) RMSE of range.

The ranges of two incident sources are fixed at $r_{1}=0.2\lambda ,r_{2}=1.4\lambda$, Figure 8 shows the capability of angular separation of three algorithms with the DOA of the first source is fixed at $\theta_{1}=20^{{}^{\circ}}$ and the DOA of the second source is varied from $24^{o}$ to $40^{o}$ with an interval of $2^{o}$ under the simulation environment of GSNR = 6 dB, α=1.5, and *L* = 1000. It is generally considered that the greater the angle separation, the smaller the influencesbetween the two incident sources, the better the estimation performance. The simulation results verify this point of view, that the greater the angle separation, the higher the probability of success and the lower of the RMSE of the angle estimation. Since the range parameter is obtained on the basis of the estimated DOA, thence, the performance of the range estimation has little to do with the angle separation. Under the same angle separation condition, since the SOCSR algorithm is not resilient to impulsive noise, it realizes the localization at a lower probability of success and a higher RMSE relative to the FLOCSR and NTCSR algorithm which can effectively suppress the impulse noise.

### 5.6. Simulation 6

**Figure 9:**
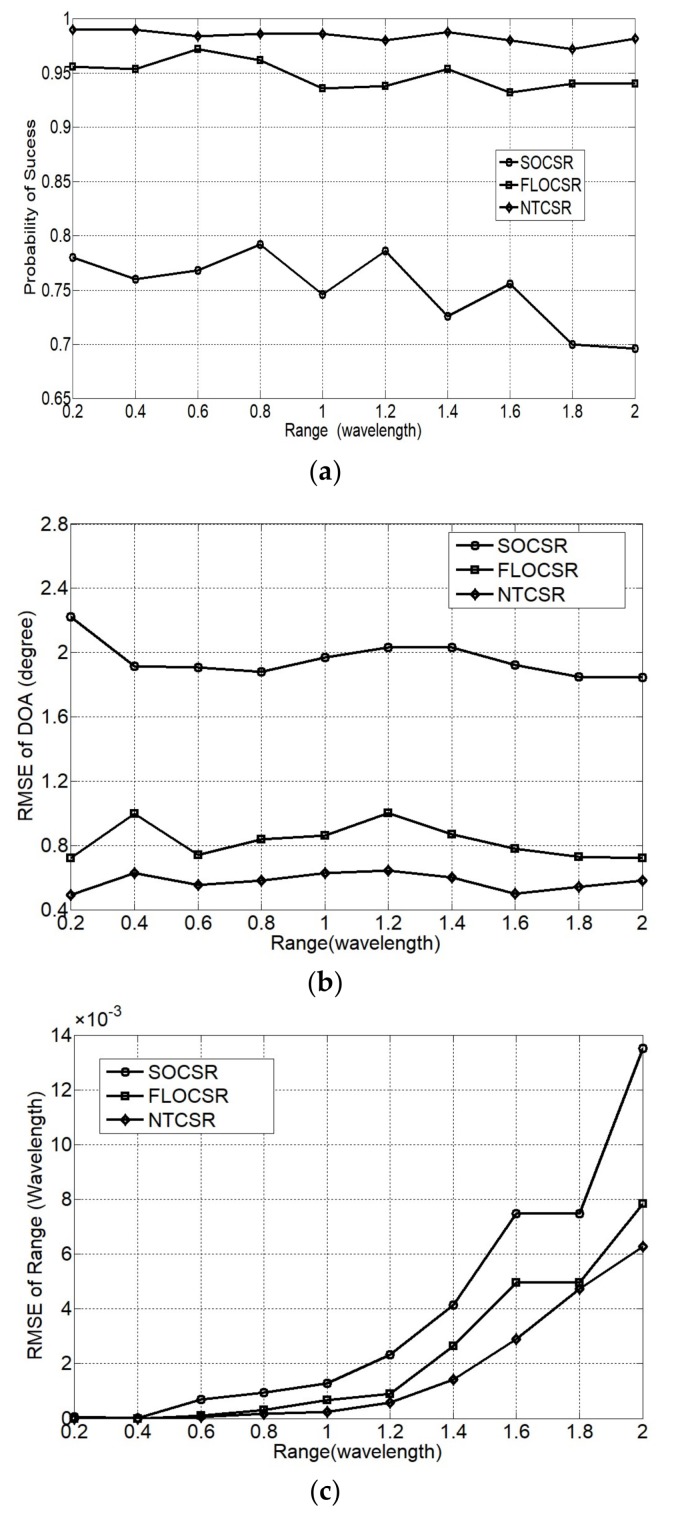
Performance as a function of range separation (**a**) probability of success; (**b**) RMSE of DOA; and (**c**) RMSE of range.

To test the capability of range separation of the algorithms, we fix the directions of two incident sources at $\theta_{1}=20^{{}^{\circ}},\theta_{2}=40^{{}^{\circ}}$ and the range of the first source at $r_{1}=0.2\lambda$. The range of the second source is varied from $r_{2}=0.2\lambda$ to $r_{2}=2\lambda$ with an interval of 0.2λ. The plots in Figure 9 are obtained under GSNR = 6 dB, α=1.5 and *L* = 1000. According to the principle of the algorithm, DOA estimation is independent of range, accordingly, the range separation changes will not affect the estimation results of DOA. Therefore, as shown in Figure 9, the probability of success and the RMSE of DOA hardly vary with range separation. However, the RMSE of range improves with the increase of range separation. In any case, our proposed two algorithms have better probability of success and lower RMSE than the SOCSR algorithm.

### 5.7. Simulation 7

**Figure 10:**
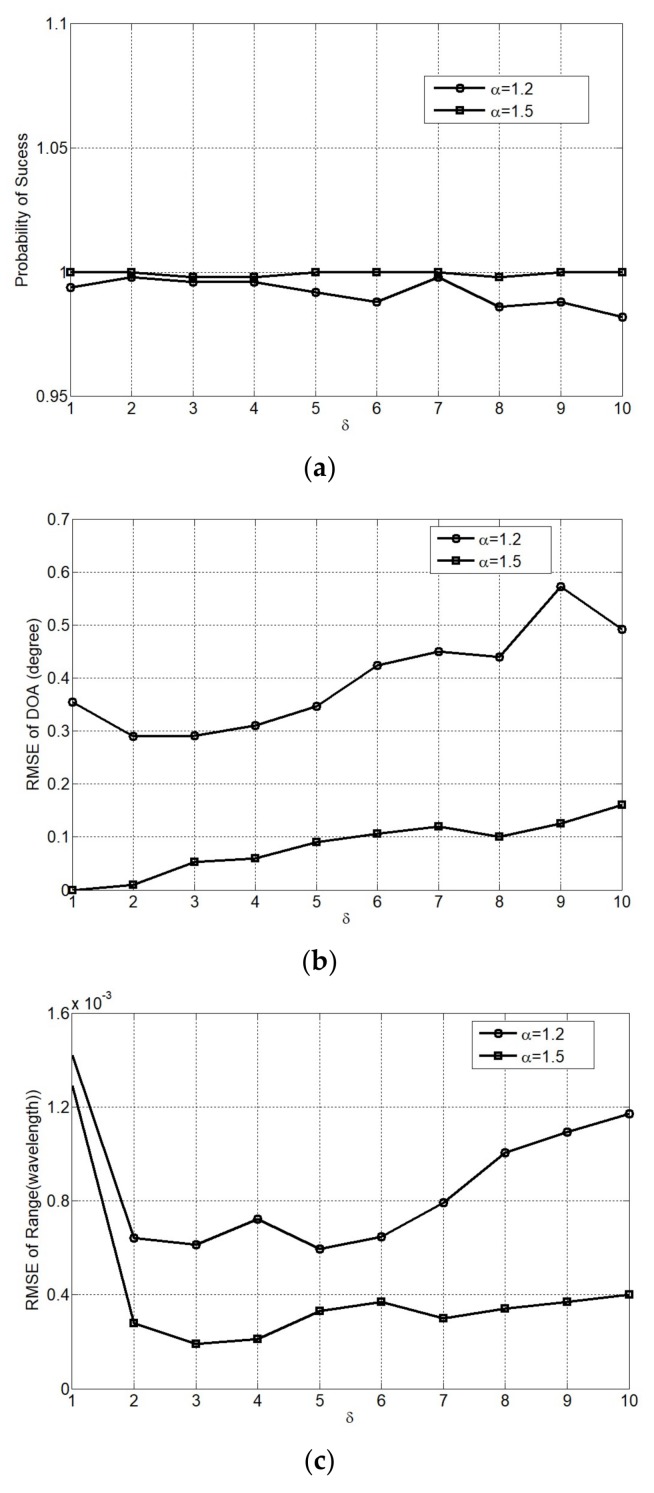
Performance of NTCSR algorithm as a function of scale factor (**a**) probability of success; (**b**) RMSE of DOA; and (**c**) RMSE of range.

In the NTCSR algorithm, the scale factor δ determines the degree of nonlinear transformation of the array received signals, in other words, it determines the degree of the impulse noise suppression. Figure 10 shows the performance of NTCSR algorithm in α = 1.2 and 1.5 two different impulse noise conditions for various scale factors ranging from 1 to 10. The number of snapshots is *L* = 1000 and GSNR = 10 dB. Although the localization of the near-field signal sources can be relatively successfully implemented in both impulse noise environments, different scale factors result in different estimation accuracies. From Figure 10 we can observe that δ∈[3–5] would be the optimal domain for NTCSR algorithm to achieve its best performance in the RMSE of DOA and range.

## 6. Conclusions

In this paper, the localization problem of near-field sources under impulsive noise environments is studied. Based on the symmetrical characteristic of the array, we construct the robust far-field approximate correlation vector in relation with the DOA only, which allows for bearing estimation based on the sparse reconstruction of the robust correlation vector. With the estimated bearing, the range can consequently be obtained by the sparse reconstruction of the output of a virtual array. Simulation results indicate the superiority of the presented two algorithms in the probability of success and RMSE under a variety of impulsive noise environments.
